# Ru-Ce_0.7_Zr_0.3_O_2−δ_ as an Anode Catalyst for the Internal Reforming of Dimethyl Ether in Solid Oxide Fuel Cells

**DOI:** 10.3390/nano14070603

**Published:** 2024-03-28

**Authors:** Miguel Morales, Mohammad Rezayat, Sandra García-González, Antonio Mateo, Emilio Jiménez-Piqué

**Affiliations:** 1 Structural Integrity and Materials Reliability Centre (CIEFMA), Department of Materials Science and Engineering, EEBE—Campus Diagonal Besòs, Universitat Politècnica de Catalunya—BarcelonaTech, C/Eduard Maristany, 16, 08019 Barcelona, Spain; mohammad.rezayat@upc.edu (M.R.); sandra.garcia@upc.edu (S.G.-G.); antonio.manuel.mateo@upc.edu (A.M.); emilio.jimenez@upc.edu (E.J.-P.); 2 Barcelona Research Center in Multiscale Science and Engineering, EEBE—Campus Diagonal Besòs, Universitat Politècnica de Catalunya—BarcelonaTech, C/Eduard Maristany, 16, 08019 Barcelona, Spain

**Keywords:** solid oxide fuel cells (SOFCs), dimethyl ether (DME), anode catalyst layer (ACL), doped ceria, partial oxidation, carbon deposition

## Abstract

The development of direct dimethyl ether (DME) solid oxide fuel cells (SOFCs) has several drawbacks, due to the low catalytic activity and carbon deposition of conventional Ni–zirconia-based anodes. In the present study, the insertion of 2.0 wt.% Ru-Ce_0.7_Zr_0.3_O_2−δ_ (ruthenium–zirconium-doped ceria, Ru-CZO) as an anode catalyst layer (ACL) is proposed to be a promising solution. For this purpose, the CZO powder was prepared by the sol–gel synthesis method, and subsequently, nanoparticles of Ru (1.0–2.0 wt.%) were synthesized by the impregnation method and calcination. The catalyst powder was characterized by BET-specific surface area, X-ray diffraction (XRD), field emission scanning electron microscopy with an energy-dispersive spectroscopy detector (FESEM-EDS), and transmission electron microscopy (TEM) techniques. Afterward, the catalytic activity of Ru-CZO catalyst was studied using DME partial oxidation. Finally, button anode-supported SOFCs with Ru-CZO ACL were prepared, depositing Ru-CZO onto the anode support and using an annealing process. The effect of ACL on the electrochemical performance of cells was investigated under a DME and air mixture at 750 °C. The results showed a high dispersion of Ru in the CZO solid solution, which provided a complete DME conversion and high yields of H_2_ and CO at 750 °C. As a result, 2.0 wt.% Ru-CZO ACL enhanced the cell performance by more than 20% at 750 °C. The post-test analysis of cells with ACL proved a remarkable resistance of Ru-CZO ACL to carbon deposition compared to the reference cell, evidencing the potential application of Ru-CZO as a catalyst as well as an ACL for direct DME SOFCs.

## 1. Introduction

Solid oxide fuel cells (SOFCs) efficiently and cleanly convert chemical energy into electrical power. They can be used in a wide range of applications, from small portable systems to large-scale power plants, with power outputs ranging from just a few watts to several megawatts [1,2]. In recent decades, great interest has been garnered by the advancements of SOFCs in portable and transport sectors, such as small auxiliary power units (APUs) and portable power generation stations [3,4]. In these types of applications, the use of high-energy-density liquid fuels is more recommended than hydrogen or natural gas since it simplifies the system by eliminating the fuel purification and reforming units. Compared with hydrogen, oxygenated hydrocarbon fuels generally have higher energy density and availability and are easier to transport and store [5,6,7]. Among oxygenated hydrocarbon fuels, dimethyl ether (DME) presents particularly interesting properties like a high hydrogen-to-carbon ratio, no carbon–carbon bond, and consequently a lower reforming temperature and a non-toxic and non-corrosive nature [8]. In addition, DME production costs have been significantly decreased in the last decade, due to the advancement of a more direct and efficient synthesis method for hydrogen and carbon monoxide or carbon dioxide, which could be categorized as a renewable fuel [9,10,11]. It can be in a liquid state at room temperature at a low pressure (~4 atm), with physical properties close to liquefied petroleum gases (LPG), probably allowing the adoption of LPG infrastructure for DME [12]. Therefore, DME may be easily transported and fed directly into SOFCs, without external reforming units, which is desirable for portable and transport applications. 

The conventional Ni-based anodes of state-of-the-art SOFCs present excellent electronic and ionic conductivity and considerable catalytic activity in both fuel cell and electrolyser modes using H_2_ and water [13]. However, several drawbacks are associated with carbon deposition for the production of synthesis gas (H_2_ and CO mixtures) using the typical reforming methods of alcohols and hydrocarbons such as steam reforming (SR), partial oxidation (PO), and auto-thermal reforming (ATR) [6,14,15]. In particular, DME PO is an attractive option for SOFC-feeding applications since this process presents a remarkable catalytic activity even at temperatures lower than 700 °C, using supported noble metal catalysts such as Rh and Pt [16,17,18]. The reaction of DME PO can be expressed by Equation (1):

(1) CH32O+12O2→3H2+2CO ∆H°=−37 kJmol−1

Although DME PO requires a lower operation temperature and exhibits a higher resistance to carbon deposition, due to its exothermic nature and oxidizing character, Ni-based anodes operating under DME and O_2_ mixtures present problems related to carbon deposition particularly at temperatures lower than 700 °C [19,20]. The state-of-the-art SOFC complies with several approaches for improving the Ni-based anode performance when running on hydrocarbon and oxygenated hydrocarbon fuels at low temperatures [21,22,23]. Recently, the addition of metal promoters [24,25] and the infiltration or ex-solution of nanoparticles [24,26,27] at the anode, the insertion of anode catalyst layer or anode functional layer [28,29], and microstructure tuning [26,30,31] have been proposed for low- and intermediate-temperature SOFCs. The insertion of an anode catalytic layer (ACL) between the anode surface and the gas supply is a promising alternative since it can protect the anode from coking and enhance the internal reforming activity. In addition, an ACL presents some advantages such as no modification of anode support is required in the original SOFC and the microstructure and thickness of ACL can be easily controlled by coating techniques, particle size, the composition of starting powders, and organic amounts. However, few studies have reported the use of ACLs for the direct reforming of DME and air mixtures in SOFCs. Firstly, Hibino et al. [18] investigated several metal/SDC/Ni ACLs deposited onto the Ni-SDC anode surface. The ACLs were prepared by mixing each metal oxide (PdO, PtO_2_, Rh_2_O_3_, or RuO_2_) with SDC and NiO powders, deposited onto anode surface, and sintered at 1380 °C. According to the values of open circuit voltage and ohmic/electrode resistances (at ~430 °C and DME:O_2_ = 1.8:1) in single-chamber SOFC configuration, the best cell performance was reached using a 5 wt.% Ru/SDC/Ni ACL. It was mainly attributed to the increase in DME PO catalytic activity at the ACL. However, its resistance to carbon deposition was not reported in detail. Later, Su et al. [32] investigated the direct DME–air reforming at intermediate temperatures using a Pt/Al_2_O_3_-Ni/MgO ACL deposited onto the Ni-YSZ anode surface for a cell with YSZ electrolyte and BSCF-SDC cathode. The ACL sintered at 850 °C exhibited a high catalytic activity for DME PO at 700 °C and DME:O_2_ = 2:1 and also a much higher resistance to carbon deposition than the Ni-YSZ anode. These good properties were attributed to the lower acidity, the lower nickel amount, and the lower sintering temperature of Pt/Al_2_O_3_-Ni/MgO ACL than those of the Ni-YSZ anode. 

Considering the importance of composition and microstructure on the catalytic properties of ACLs and the resistance to carbon formation, new advances in DME PO catalysts for syngas production open avenues to implement its improvements to ACLs. A review of the state of the art on the DME PO catalysts showed that Wang et al. [16] first reported DME PO activities of metals supported on Al_2_O_3_ to be much higher than those supported on MgO and La_0.8_Sr_0.2_Ga_0.8_Mg_0.15_Co_0.05_O_3_ (LSGMC). At low temperatures, the syngas production decreases in the order of metals (supported on Al_2_O_3_): Rh, Ni, Co, Ru, Fe, Pt, and Ag. However, carbon formation is a common problem for all the mentioned catalysts, which is associated with high methane production [33]. Later, some researchers demonstrated that combining mixtures of different metals and supports is a good solution to obtain a high DME PO conversion, with concentrations > 90% of hydrogen and <10% of methane. For instance, Zhang et al. [34] reported a study of DME PO using Pt/Al_2_O_3_ and Ni-MgO, achieving an high hydrogen yield (>90%) combined with a low methane production. Yu et al. [35] and Kim et al. [36] proposed other alternatives based on 0.5 wt.% Rh/γ-Al_2_O_3_/Al and 0.05 wt.% Rh/γ-Al_2_O_3_/FeCrAl, respectively, which achieved high DME conversion (>85%), a complete O_2_ conversion, and >85% H_2_ selectivity at 450 °C. The Rh/γ-Al_2_O_3_/FeCrAl catalyst maintained a high performance for 1200 h. More recently, Badmaev et al. [37,38] have reported studies of noble metal/Ce_0.75_Zr_0.25_O_2_ (noble metal = Pt, Rh, Ru) catalysts prepared via sorption–hydrolytic deposition, which exhibits a high performance for DME PO. Zirconium-doped ceria (CZO), as a support, provides a high dispersion of noble metal and high resistance to carbon deposition, due to a strong metal–support interaction and excellent oxygen mobility of CeO_2_-based materials [39]. The high oxygen storage capacity (OSC) of Ce_1−x_Zr_x_O_2_ is observed for compositions of 0.2 < x < 0.6. On the other hand, Pt, Rh, and Ru, as noble metals, have a good ability to catalyse the breaking of C-C bonds and a very low affinity to form both carbides and carbon nanofibers. Therefore, it suggests that the insertion of noble metal/ceramic, as an ACL, for the direct DME PO in SOFCs may be a promising candidate. Hence, this solution was successfully explored for low-temperature SOFCs operated on different mixtures of hydrocarbons (methane, iso-octane, etc.) and air or water [18,40,41].

In the present study, the insertion of 2.0 wt.% Ru-Ce_0.7_Zr_0.3_O_2−δ_ (ruthenium–zirconium-doped ceria, Ru-CZO) as an ACL is explored, for the first time, as an alternative for the internal reforming of dimethyl ether in SOFCs. This alternative could present an interesting performance and be cost-effective, as Ru and CZO are cheaper than other catalysts containing precious metals such as Pt and Rh and supports based on gadolinium or samarium-doped ceria. For this purpose, the CZO powder was prepared by the sol–gel synthesis method, and subsequently, nanoparticles of Ru (1.0–2.0 wt.%) were synthesized by the impregnation method and calcination. The quality of the synthesized catalyst precursor was characterized by BET-specific surface area, X-ray diffraction (XRD), scanning electron microscopy (SEM), and transmission electron microscopy (TEM) techniques. Afterward, the catalytic activity of Ru-CZO catalysts was studied under DME PO. Finally, button anode-supported SOFCs with Ru-CZO ACL were prepared, depositing Ru-CZO onto the anode support and using an annealing process. The effect of Ru-CZO ACL on the electrochemical performance of the cell was investigated under a DME and air mixture at 750 °C and was compared with a reference cell without ACL.

## 2. Experimental Procedure

### 2.1. Materials Synthesis

Zirconium-doped ceria (Ce_0.7_Zr_0.3_O_2−δ_, CZO) was synthesized using the EDTA–citrate sol–gel method. The aqueous solution of Ce(NO_3_)_3_·6H_2_O (Sigma-Aldrich, St. Louis, MO, USA, 99%) and ZrO(NO_3_)_2_·xH_2_O (Sigma-Aldrich, 99%) in stoichiometric proportions was continuously stirred at 60 °C. After the evaporation of solvent at 80 °C, the gel was calcined at 500 °C for 5 h in air to form the precursor CZO powder. Afterward, Ru-Ce_0.7_Zr_0.3_O_2−δ_ powders with nominal compositions of 1.0, 1.5, and 2.0 wt.% Ru were prepared via the wet impregnation method. An appropriate amount of CZO was impregnated under continuous stirring at 80 °C in a solution of Ru(NO)(NO_3_)_3_ (Alfa Aesar, Haverhill, MA, USA, Ru 1.5% *w*/*v*). Finally, the samples were dried at 120 °C in air and calcined at 500 °C for 2 h in air. The detailed descriptions of the above synthesis procedures can be found in a previous study [42].

### 2.2. Catalyst Characterization

The specific surface area of the catalysts was determined by the Brunauer–Emmett–Teller method using a Micromeritics model Tristar 3000 (Norcross, GA, USA). The measurements were performed through nitrogen adsorption at 77 K. Ru-CZO was analysed by X-ray diffraction (XRD, Bruker, D8-Advance, Billerica, MA, USA) using Cu Kα radiation (operated at 40 kV and 40 mA). The data collection was carried out at room temperature, between 20° and 80°, with a step size of 0.01° and a collection time of 1 s/step. Phase identification was performed using the JCPDS database and the DIFFRACplus EVA software (V7) by Bruker AXS. The crystallite size of the CZO and Ru-CZO was calculated by the line-broadening analysis according to the Scherrer equation. The catalyst and fuel cell microstructures were observed by scanning electron microscopy (SEM; Carl Zeiss Merlin, Jena, Germany) equipped with an energy-dispersive spectroscopy detector (EDS; Oxford Instruments INCA-350 system, Abingdon, UK). The microstructure of the CZO powder was analysed by transmission electron microscopy (TEM; JEOL 1210 TEM, Tokyo, Japan) operating at 120 kV accelerating voltage. A sample for TEM analysis was prepared by dispersing the nanoparticles in ethanol and then drop cast on a TEM copper grid. To determine carbon formation under DME–air, the fuel cells were analysed using Raman spectroscopy (inVia Qontor, Renishaw, Dundee, IL, USA). Two lasers with distinct wavelengths of the applied excitation line were used in the infrared region (785 nm) and in the visible region (532 nm). An optical microscope with a 100× objective was employed to determine the analysis zone. The surface properties related to the chemical states and surface compositions of Ce, Zr, and O in the CZO samples were determined by X-ray photoelectron spectroscopy (XPS). XPS analyses were conducted in an ultrahigh-vacuum multichamber system by SPECS with a PHOIBOS 150 EP hemispherical energy analyzer and an MCD-9 detector XR-50. It possesses an X-ray source with a twin anode (Al and Mg) and a high-pressure and high-temperature chamber for gas treatments of the samples. The samples were compensated for charging with a low-energy electron beam, and the peak of C 1s (binding energy = 284.4 eV) was used to correct sample charging effects [43]. SpecsLab Prodigy (Version 4.113.1), an experiment control software package, was used for data acquisition and CasaXPS (2.3.25) for spectral analysis.

### 2.3. Catalytic Tests

The study of the Ru-CZO catalytic activity under DME and air mixtures was carried out in a fixed-bed quartz tubular reactor (5 mm inner diameter) at atmospheric pressure. A 50 mg quantity of Ru/CZO was packed on a bed of quartz wool in the reactor, which was kept in a horizontal tubular furnace. Two K-type thermocouples were used, i.e., one outside the reactor to control the furnace temperature using a Eurotherm PID controller and another in contact with the catalyst to control its temperature. Before catalyst tests, several blanks (without a catalyst) were analysed to confirm the absence of direct oxidation reactions in the reactor. Before the partial oxidation of the DME reaction, the catalyst precursor was reduced at 750 °C for 1 h in 5 vol% H_2_/Ar (30 mL min^−1^) and then cooled under N_2_ (30 mL min^−1^) to the initial testing temperature of 300 °C. The effect of the temperature on the DME conversion and the selectivity of H_2_, CO, CO_2_, and CH_4_, over Ru-CZO, was analysed under the following operation parameters: DME:O_2_ = 2:1 (vol%); DME:O_2_:N_2_ = 30:15:55 (vol%); and GHSV = 10,000 h^−1^. The outlet gas of catalysts was analysed using online gas chromatography (Agilent Micro GC 3000, Santa Clara, CA, USA). The additional details of the experimental setup can be found in a previous study [42]. The DME conversion and the selectivity of H_2_ and C-containing products (CO, CO_2_, and CH_4_) were calculated according to the following Equations (2)–(4):

(2) $$DMEconv\left(\%\right)=\frac{{\left(DME\right)}_{\left(mmol/min,in\right)}-{\left(DME\right)}_{\left(mmol/min,out\right)}}{{\left(DME\right)}_{\left(mmol/min,in\right)}}\times 100$$

(3) $$H_{2}selectivity\left(\%\right)=\frac{{\left(H_{2}\right)}_{\left(mmol/min,out\right)}}{{3\times \left(DME\right)}_{\left(mmol/min,in\right)}}\times 100$$

(4) $$C_{n}selectivity\left(\%\right)=\frac{n\times C_{\left(n,mmol/min,out\right)}}{{2\times \left(DME\right)}_{\left(mmol/min,in\right)}}\times 100$$

### 2.4. Electrochemical Tests of Fuel Cells

**Figure 1:**
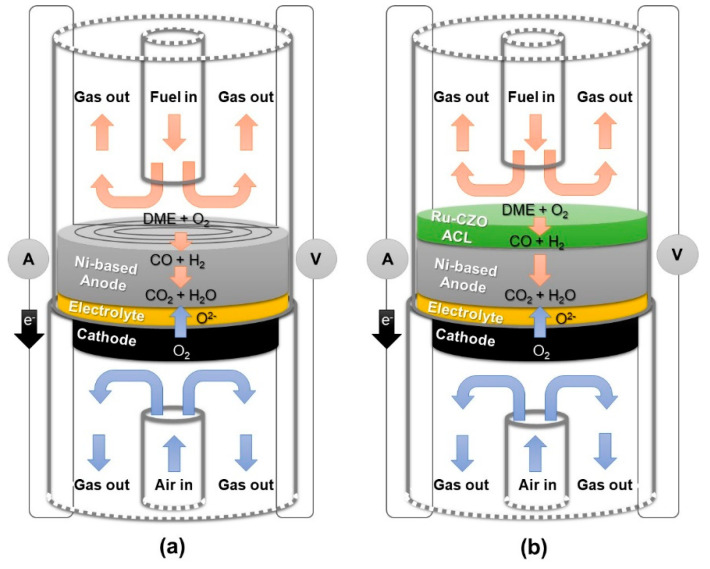
Scheme of the electrochemical test for (**a**) a reference fuel cell and (**b**) a fuel cell with ACL.

Button state-of-the-art anode-supported cells consisting of Ni-YSZ anode, YSZ electrolyte, CGO barrier layer, and LSCF-CGO cathode, with ACL (ACL cell) and without ACL (reference cell) were tested in a homemade test bench able to measure button cells (Figure 1). Before tests, NiO and LSC paste current collecting layers were painted on the sides of the anode and cathode surfaces of cells, respectively, and annealed at 800 °C in air for 2 h. Electrical connections were made using four Pt wires, and Pt meshes were used as the current collectors. Afterward, the cells were sealed on two alumina tubes using a Ceramabond sealant and reduced in a humidified H_2_ atmosphere at 800 °C for 2 h. In the case of a cell with ACL, colloidal Ru-CZO paste with terpineol (1:5 wt/wt) was painted on the surface of an anode-current collector and annealed at 800 °C in air for 2 h. After the reduction process, the characteristic *j*-V (current density–voltage) curves were collected for H_2_ (40 mL·min^−1^) and DME:O_2_:N_2_ = 30:15:55 (vol%) at 750 °C. The flow rate of synthetic air in the cathode was fixed at 300 mL min^−1^. The reaction gases were supplied to the reactor using mass flow controllers. A K-type thermocouple was fixed to the anode to determine the cell temperature. The characteristic *j*-V curves of the cells were determined by a sourcemeter unit (Keithley 2420, Cleveland, OH, USA) using a four-probe configuration. The outlet gas of the cell anode was analysed using online gas chromatography (Agilent Micro GC 3000). The additional details of the experimental setup can be found in a previous study [28].

## 3. Results and Discussion

### 3.1. Characterization of Ru-CZO Powders

**Figure 2:**
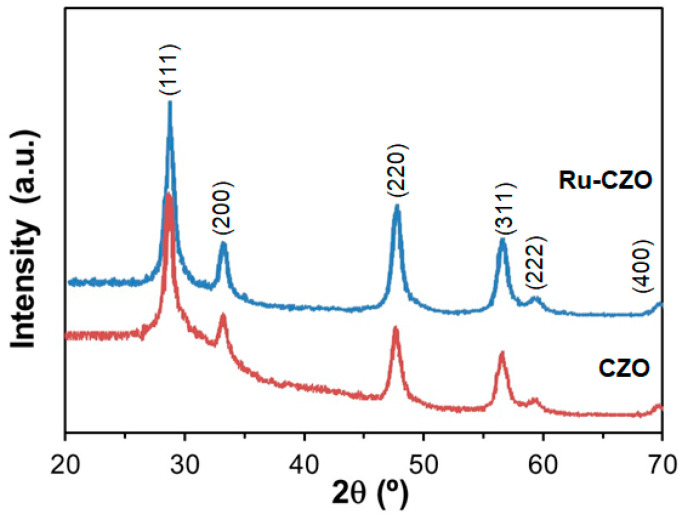
X-ray diffraction (XRD) patterns of the Ce_0.7_Zr_0.3_O_2−δ_ powders, after calcining at 800 °C, in which are marked the corresponding Miller indexes for the interplanar spacing values of the face-centred cubic fluorite-type phase of CeO_2_ (JCPDS 34–0394).

Figure 2 shows the XRD patterns of the CZO and Ru-CZO powders, after calcining at 800 °C, which presented an S_BET_ of 42 and 46 m^2^/g, respectively. The results suggested that no difference in the characteristic diffraction peaks of Ru-CZO and CZO was observed, which were indexed to the fluorite-type phase of ceria (JCPDS 34–0394) [44]. Therefore, the CZO remains unchanged after the impregnation of Ru cations. There is no detected peak attributed to ruthenium nanoparticles, due to the low ruthenium amount on the CZO. In addition, no extra peak in the XRD patterns of the samples attributed to impure phases was detected. The crystallite size of CZO from the XRD data was about 120 Å.

**Figure 3:**
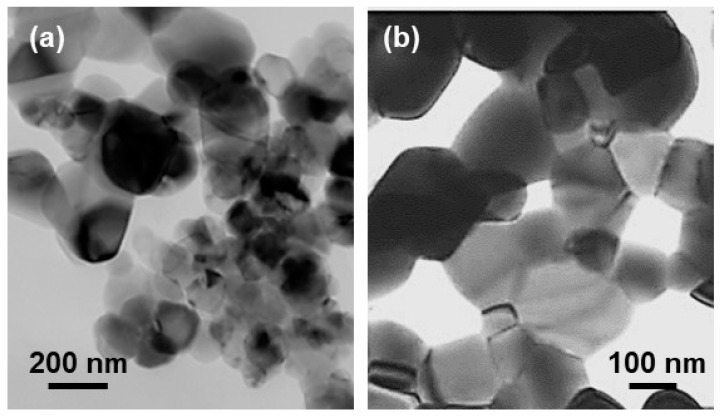
TEM images of CZO particles at: (**a**) low and (**b**) high magnification.

**Figure 4:**
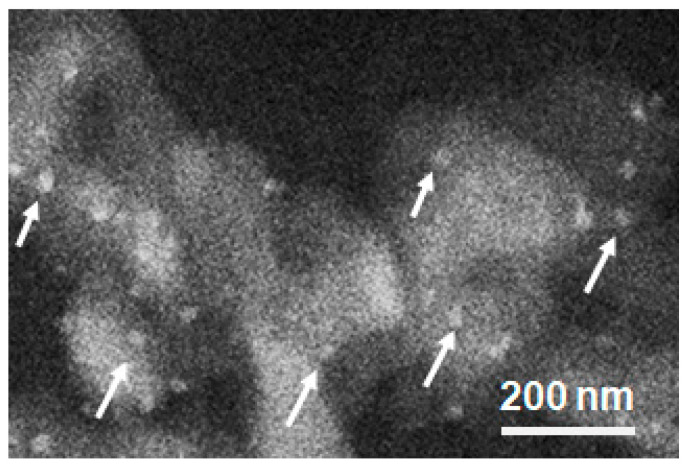
FESEM image of Ru-CZO obtained with backscattered electron detector. The white arrows show some of the Ru nanoparticles dispersed on the CZO surface.

**Figure 5:**
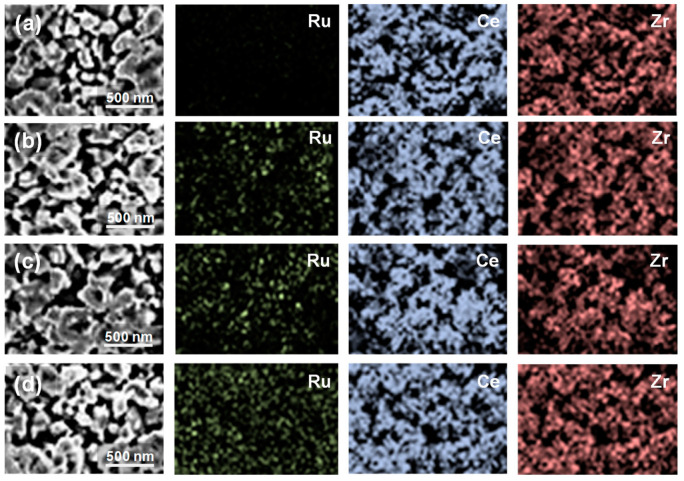
FESEM-EDS elemental mapping of Ru, Ce, and Zr for: (**a**) CZO and (**b**) 1.0, (**c**) 1.5, and (**d**) 2.0 wt.% Ru-CZO.

The microstructure and compositional distribution of fresh Ru-CZO powder were characterized by FESEM-EDS and TEM. The TEM images of the CZO exhibited a spherical morphology and particle size in the range between 100 and 200 nm (Figure 3). Figure 4 exhibits the FESEM images of Ru-CZO. A random dispersion of Ru nanoparticles on the CZO was observed by the FESEM backscattered electron detector (Figure 4). It suggested that Ru was highly distributed along the entire active area of the CZO. The size of spherical Ru nanoparticles was estimated to be between 20 and 30 nm. Figure 5 shows the representative FESEM-EDS images of CZO and Ru-CZO. These images confirmed the homogeneous composition of Ce and Zr. In addition, the Ru was uniformly distributed in the CZO (Figure 5b–d). In Table 1, the average values (in wt.%) of the constituent elements for each sample, which were determined from at least six points, are presented to confirm the chemical compositions. The obtained compositions were close to the theoretical values, which were within the typical errors associated to FESEM-EDS analysis.

**Figure 6:**
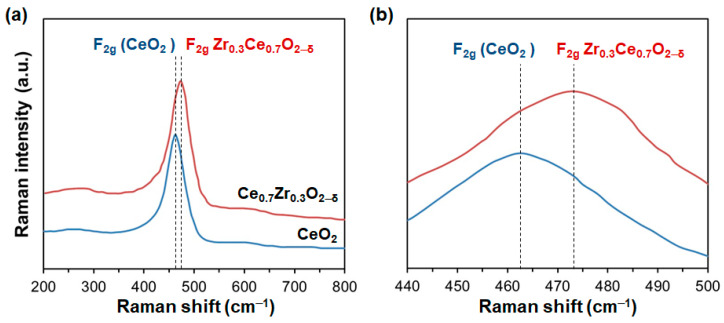
Raman spectra of the Ce_0.7_Zr_0.3_O_2−δ_ and CeO_2_ as a reference in (**a**) the region of 200–800 cm^−1^ and (**b**) the ceria peak region.

Raman spectroscopy was carried out to complement the XRD analysis of CZO powders. Figure 6 shows the Raman spectra of the CZO sample and CeO_2_ as a reference. Undoped ceria presented the first-order Raman peak at ~462 cm^−1^, which was assigned to the F_2g_ Raman active mode of a fluorite-structured material [45]. For the CZO sample, the intense absorption band was centred at ~474 cm^−1^, which shifted to a higher wavenumber due to the increase in compressive stress in the sample. In addition, weak bands at ~250 and ~600 cm^−1^ were also observed in the CZO. The weak peak at 250 cm^−1^ could be related to the distortion of the cubic lattice, which produced a lattice compression and the corresponding lattice stress. Meanwhile, the adsorption band at 600 cm^−1^ was associated with the oxygen vacancy of CeO_2_ [46]. Therefore, the CZO presented a major surface concentration of oxygen vacancies. In addition, a solid solution was formed in CZO, as no Raman adsorption band and XRD peak induced by ZrO_2_ were detected. It can be concluded that a certain content of oxygen defects could play a remarkable role in DME catalysis. 

**Figure 7:**
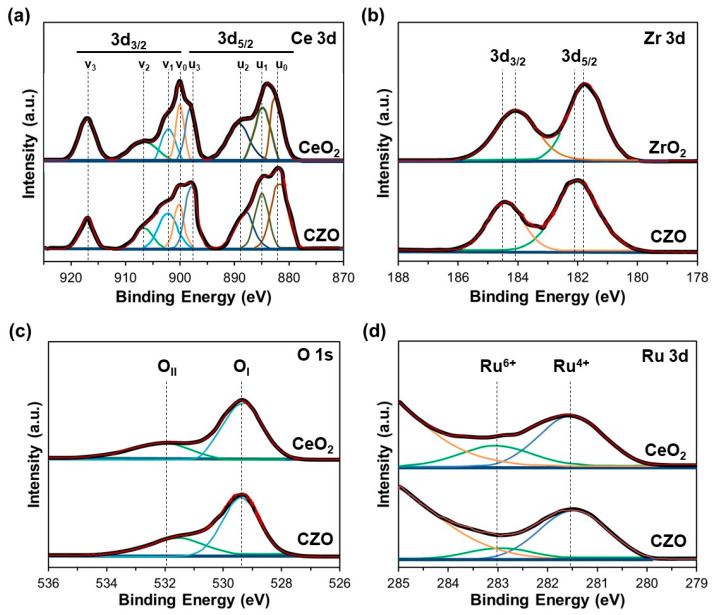
XPS spectra of (**a**) Ce 3d, (**b**) Zr 3d, (**c**) O 1s, and (**d**) Ru 3d.

On the other hand, the surface properties related to the chemical states, surface compositions, and adsorbed species in the as-prepared Ru-CZO sample were studied by X-ray photoelectron spectroscopy (XPS). Additionally, these XPS results were compared with Ru-CeO_2_ and ZrO_2_ to determine the improvements in CZO as a support of Ru. Figure 7 shows the XPS spectra of Ce 3d, Zr 3d, O 1s, and Ru 3d. In Figure 7a, the 3d_3/2_ and 3d_5/2_ spin-orbital components of the Ce 3d were denoted with *u* and *v*, respectively. Among these bands, *u*_0_ (882.1 eV), *u*_2_ (888.9 eV), and *u*_3_ (897.6 eV) were the characteristic bands of 3d_3/2_ spin-orbital components of Ce^4+^, whereas *v*_0_ (900.2 eV), *v*_2_ (907.2 eV), and *v*_3_ (916.4 eV) were assigned to the 3d_5/2_ spin-orbital components of Ce^4+^. The *u*_1_ (884.7 eV) and *v*_1_ (902.2 eV) bands were attributed to the emissions of 3d_3/2_ and 3d_5/2_ spin-orbital of Ce^3+^, respectively [47,48,49,50]. The percentage of Ce^3+^ in the CeO_2_ and CZO was determined from the fraction of the sum of the area of total Ce^3+^ species (*u*_1_ and *v*_1_) to the sum of the area of total Ce species [47,48,49]. The CZO presented a Ce^3+^ concentration (28%) higher than CeO_2_ (21%), which is crucial for improving the catalytic properties of Ce_1−x_Zr_x_O_2_. In the case of Zr 3d spectrum (Figure 7b), the binding energies of 3d_5/2_ and 3d_3/2_ were 182.6 eV and 184.5 eV, respectively. These values of binding energy for Zr 3d in the CZO were higher than those in the ZrO_2_ phase (about 0.5–0.8 eV) [51,52], which are in good agreement with those of previous studies for the same CZO composition [53,54]. Thus, the formation of Ce-Zr solid solutions in the CZO instead of the ZrO_2_ phase is confirmed. Figure 7c shows the O 1s signals of the samples. These signals were separated into two components at 529.4 and 532.9 eV, which were attributed to the lattice oxygen (O_I_) and chemisorbed oxygen (O_II_), respectively. The band at 529.4 eV was associated with the Ce-O-Zr complex (O^2−^), whereas the band at 532.9 eV was related to O^2−^ at the surface such as O^2−^, OH^−^, CO_3_^2−^, etc. [53,55]. Thus, CZO had a slight increase in the O_II_/O_I_ peak area ratio (0.32) compared with that of CeO_2_ (0.24), which could effectively increase the concentration of surface-adsorbed oxygen. Compared to the surface lattice oxygen (O_I_), the chemically adsorbed oxygen (O_II_) presents higher mobility, which could play a remarkable role in the DME partial oxidation. Finally, Figure 7d shows Ru 3d XPS spectrum of the samples, which was partially overlapped with the C 1s XPS spectrum. Thus, it was divided into two components at 281.6 and 283.0 eV, which were assigned to the surface Ru^4+^ and Ru^6+^, respectively, and correspond to the 4f_5/2_ states [56,57,58]. In this case, the component at >285 eV was assigned to the C1s signal. No signal of Ru^0^ was detected, evidencing that all of Ru on the surface was present in an oxidized state after calcination in air. Nevertheless, the surface Ru^6+^/Ru^4+^ molar ratio of Ru-CZO was lower (0.42) than that of Ru-CeO_2_ (0.23), which suggested that the CZO could enhance the oxidation state of Ru.

### 3.2. Catalytic Tests of Ru-CZO

**Figure 8:**
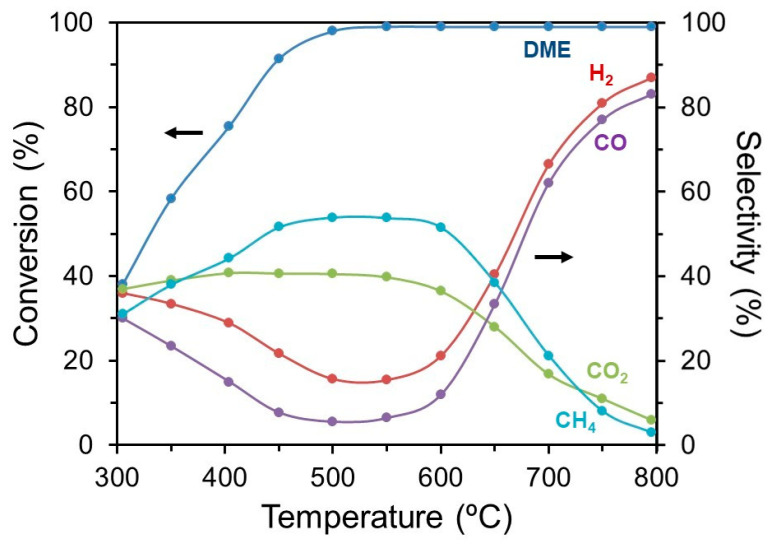
Effect of the temperature on the DME conversion and the selectivity of H_2_, CO, CO_2_, and CH_4_, over Ru-CZO. Operation parameters: DME:O_2_ = 2:1 (vol%); DME:O_2_:N_2_ = 30:15:55 (vol%); and GHSV = 10,000 h^−1^.

Figure 8 shows the effect of temperature on the DME conversion and the selectivity of the main products (H_2_, CO, CO_2_, and CH_4_) over 2.0 wt.% Ru-CZO in an inlet composition of DME:O_2_:N_2_ = 20:15:55 (vol%). DME conversion increased as a function of the temperature, achieving complete DME conversion at 550 °C. The product selectivity of H_2_, CO, CO_2_, and CH_4_ presented a complex distribution. The selectivity of CO_2_ and CH_4_ increased with temperature, reaching almost constant values between 450 and 600 °C, and above 600 °C sharply decreased, obtaining about 17% and 11% at 750 °C. Meanwhile, the H_2_ and CO presented an inverse behaviour with low selectivity between 450 and 550 °C. Above 550 °C, the H_2_ and CO selectivity strongly increased with the temperature. After catalytic tests, no formation of deposited carbon was detected by Raman spectroscopy and by measuring the weight of the sample. Thus, the Ru-CZO catalyst provided a complete DME conversion and high yields of H_2_ and CO above 700 °C. This suggests that the 2.0 wt.% Ru-CZO catalyst may supply synthesis gas from an efficient partial oxidation of DME and air, which may be potentially suitable for SOFC applications at intermediate temperatures (750–800 °C). 

To determine the effect of Ru composition on the catalytic activity of Ru-CZO, several experiments were carried out. Table 2 shows the results of the DME conversion and the selectivity of H_2_, CO, CO_2_, and CH_4_ over compositions between 1.0 and 2.0 wt.% Ru in the Ru-CZO catalysts at 750 °C. Under these conditions, the DME conversion was ~100% for all catalyst compositions, and H_2_ and CO were the major reaction products. However, the selectivity of H_2_ and CO was enhanced with the increase in the Ru amount in the catalyst, while the CO_2_ and CH_4_ selectivity was decreased. These results suggested that the ACL should possess 2.0 wt.% Ru to ensure a high selectivity towards H_2_ and CO and to minimize the CH_4_ production in the Ru-CZO catalyst layer of the SOFC.

### 3.3. Electrochemical Tests of Fuel Cells

**Figure 9:**
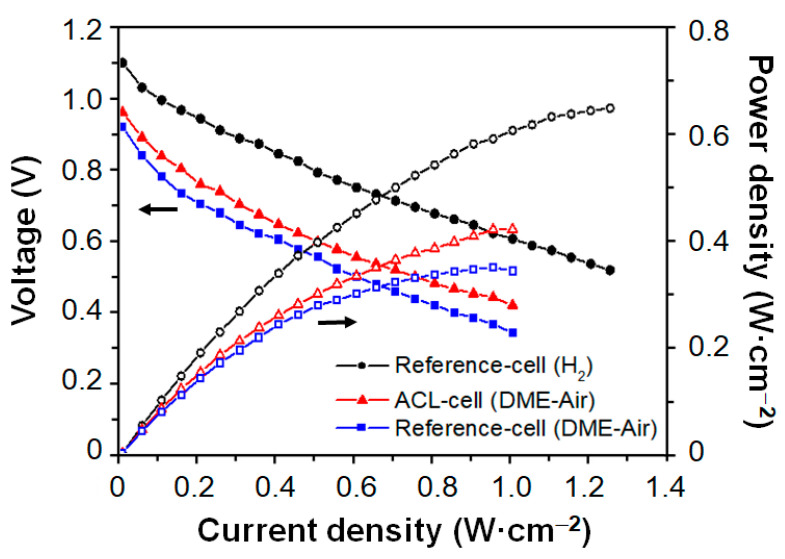
*j*-V and *j*-P polarization curves for ACL cell and reference cell at 750 °C, using 40 mL·min^−1^·cm^−2^ for humidified-H_2_ tests, and 40 mL·min^−1^·cm^−2^ DME + 90 mL·min^−1^·cm^−2^ air (DME:O_2_:N_2_ = 30:15:55 in vol%) for DME–air tests at the anode chamber and 200 mL·min^−1^·cm^−2^ synthetic air at the cathode chamber.

Two button cells with 2.0 wt.% ACL (ACL cell) and without a catalyst (reference cell) were selected to be electrochemically tested from the results of preliminary catalytic analyses. Both cells were tested at the typical operation temperature of 750 °C, under 40 mL·min^−1^·cm^−2^ humidified H_2_ and a DME–air mixture of 40 mL·min^−1^·cm^−2^ DME and 90 mL·min^−1^·cm^−2^ air (DME:O_2_:N_2_ = 30:15:55 in vol.%). Figure 9 shows the *j*-V and *j*-P polarization curves for both cells in H_2_ and DME–air. The OCV of both cells was reduced from 1.09 V to 0.96 V (ACL cell) and 0.93 V (reference cell) after fuel change from H_2_ to DME–air, respectively. It was attributed to the effect of diluted fuel (H_2_ and CO) with the presence of N_2_ and other product species (CO_2_ and H_2_O) in the anodic chamber. The power density of cells at 1.0 A·cm^−2^ was 0.42 W·cm^−2^ for the ACL cell and 0.37 W·cm^−2^ for the reference cell at 750 °C using DME–air as a feed fuel. Thus, the performance of both cells in DME–air was significantly lower than that of reference cell in H_2_ (0.61 W·cm^−2^ at 1.0 A·cm^−2^). Upon analysing the *j*-V polarization curves, it is worth mentioning that the area-specific resistances (ASRs), calculated from the slopes of the *j*-V curves, were similar in both cells using H_2_ and DME–O_2_ as fuels. 

**Figure 10:**
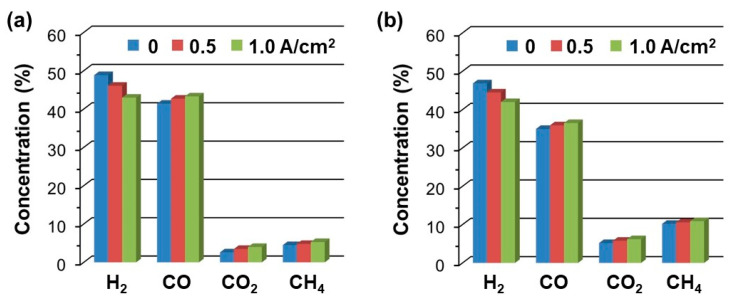
Concentration of reforming products as a function of current density for (**a**) the ACL cell and (**b**) the reference cell.

The results commented above suggested that the cell performance was mainly controlled by the fuel dilution and the DME PO-reforming activity of each cell. According to the anode exhaust compositions in Figure 10, the ACL cell presented higher concentrations in both H_2_ and CO combined with lower amounts in both CO_2_ and CH_4_ at OCV, which was in good agreement with the previous catalytic tests. The decrease in the formed CH_4_ by using the ACL is interesting to reduce the carbon deposition, as the high catalytic activity of Ni-based anodes for generating carbon nanofibers or nanotubes when directly oxidizing hydrocarbon fuels like CH_4_ [59,60,61]. Furthermore, as the applied current increased from 0 to 1.0 A·cm^−2^ in both cells, the H_2_ concentration decreased, while CO increased, and in the meantime, only a low amount of CO_2_ could be generated from the electrochemical conversion of CO. Therefore, the power output of the cell could be mainly generated from the selective oxidation of H_2_. However, O^2−^ can theoretically electro-oxidize both CO and H_2_ under the applied current. It suggested that the selective oxidation towards H_2_ may be attributed to the H_2_ electro-oxidation at the triple-phase boundary (TPB) of the Ni-based anodes of SOFCs that took place faster than that of CO, as previous studies reported [62,63,64].

### 3.4. Post-Test Analysis

**Figure 11:**
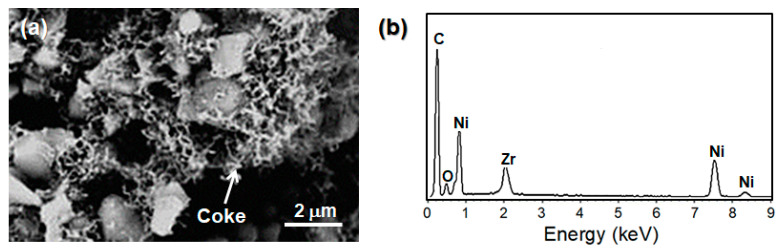
(**a**) FESEM image and (**b**) EDX spectrum of the Ni-YSZ anode corresponding to the reference cell, after operating for 3 h in the DME–O_2_ mixture gas at 750 °C.

**Figure 12:**
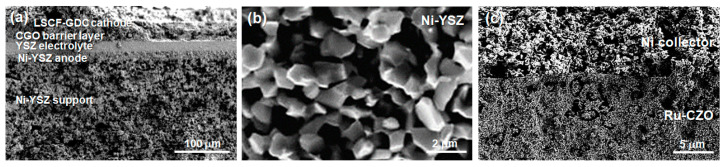
FESEM images of the ACL cell: (**a**) a cross-section view, (**b**) the Ni-YSZ anode, and (**c**) a region at the ACL/Ni-collector interface, after operating for 5 h in the DME–O_2_ mixture at 750 °C.

The post-test analysis of cells was carried out using SEM-EDS and Raman spectroscopy to evaluate the carbon deposition and microstructural damage. As shown in Figure 11, SEM-EDS analysis confirmed the carbon deposition in a filamentous morphology at the Ni-YSZ anode of the reference cell after operating under DME–air for 3 h. In addition, the anode was damaged by carbon deposition, which generated some cracks in the cell and a remarkable drop in the cell performance. In contrast, no carbon deposition was detected at the Ru-CZO ACL and the Ni-YSZ anode of the ACL cell after operating for 5 h in the DME–O_2_ mixture at 750 °C (Figure 12a–c).

**Figure 13:**
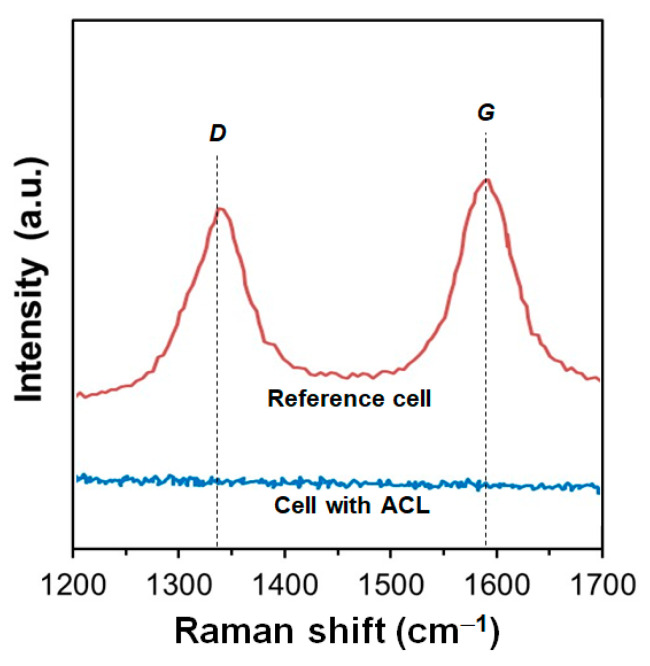
Raman spectrum in the range of 1200–1700 cm^−1^ obtained at the Ni-YSZ anode support of the ACL cell and the reference cell after testing.

Raman spectroscopy was complementarily used to determine the carbon deposition in the anode of the ACL cell and the reference cell. Figure 13 shows the Raman spectrum in the range of 1200–1700 cm^−1^ determined at the anode support of the ACL cell and reference cell. A couple of intense bands at ~1340 cm^−1^ (*D* band) and ~1590 cm^−1^ (*G* band) were observed for the reference cell, which were mainly related with the amorphous and graphite carbon phase structures [65,66]. The presence of graphite suggests that the carbon deposited on the Ni-YSZ anode of the cell without CZO is an important issue, since the elimination of graphite carbon is more difficult than that of the amorphous one. In contrast, no band was detected in the Raman spectrum for the ACL cell after testing in the DME–O_2_ mixture gas for 5 h. This suggests that no significant carbon was deposited, which is in good agreement with the SEM-EDX analyses. Thus, the resistance to the carbon formation may be improved using an ACL of 2.0 wt.% Ru/Ce_0.7_Zr_0.3_O_2−δ_. This enhancement is attributed to the synergetic effect of Ru and Ce_0.7_Zr_0.3_O_2−δ_, which could provide a strong metal–support interaction with atomically dispersed Ru and high oxygen mobility of support [39]. It is important to add that the ACL protects the carbon deposition in the anode when ACL is free of defects and perfectly covers the entire surface of the Ni-collector/Ni-YSZ-anode. Thus, those ACL cells with large defects or discontinuities in the ACL or with delamination at the ACL/Ni-collector interface also presented carbon deposition. However, this may be a challenge during cell operation, particularly when the fuel composition and current density are changed, as these can generate thermal gradients and thermo-mechanical stress in the cell, due to the mismatch of thermal expansion coefficients (TECs) between the catalyst and anode materials. For this issue, some researchers have developed an independent-catalyst layer formed of an ACL and its mechanical support near the anode for implementation [67,68].

## 4. Conclusions

The insertion of Ru-Ce_0.7_Zr_0.3_O_2−δ_ as an ACL for the internal reforming of DME PO in SOFCs was investigated as a promising strategy to mitigate carbon deposition. CZO powder was prepared by the sol–gel synthesis method, and subsequently, the nanoparticles of Ru (1.0–2.0 wt.%) were synthesized by the impregnation method and calcination. 

XRD, XPS, and Raman spectroscopy confirmed the Zr_0.3_Ce_0.7_O_2−δ_ solid solution formed by adding Zr into CeO_2_, thus inducing a lattice strain in the ceria lattice due to an increase in the oxygen defect concentration compared with CeO_2_. SEM-EDS, and TEM evidenced a high dispersion of Ru in the zirconium-doped ceria. 

Among the Ru-CZO compositions, one with 2.0 wt.% Ru provided a complete DME conversion and high yields of H_2_ and CO at 750 °C, which is the typical operation temperature of the state-of-the-art SOFCs, suggesting its potential use for SOFC applications at intermediate temperatures.

The insertion of 2.0 wt.% Ru-CZO ACL significantly increased both the OCV and the performance of the cell by more than 20%, due to the enhancement of catalytic activity towards DME PO compared with the Ni-YSZ anode. In addition, the post-test analysis of ACL cell proved a remarkable resistance of Ru-CZO ACL to carbon deposition compared with the reference cell.

Overall, the present study demonstrates that the Ru-Ce_0.7_Zr_0.3_O_2−δ_ may be a good approach for the development of a catalyst layer for the internal reforming of SOFCs directly operated on mixtures of DME and air at intermediate temperatures. Future research should be focused on the implementation of Ru-CZO in an independent active catalyst layer to avoid the possible damage associated with the thermal gradients, and its potential application with longer-term tests and a detailed post-test analysis.

## Figures and Tables

**Table 1:** Elemental compositions (in wt.%) of Ru, Ce, Zr, and O for the CZO and Ru-CZO samples determined from the FESEM-EDS analysis.

Sample	Nominal Composition Ru (wt.%)	Ru (wt.%)	Ce (wt.%)	Zr (wt.%)	O (wt.%)
CZO	0	0	62.3	18.3	19.4
1.0 wt.% Ru-CZO	1.0	1.2	61.9	18.1	18.8
1.5 wt.% Ru-CZO	1.5	1.8	61.2	18.0	19.0
2.0 wt.% Ru-CZO	2.0	2.3	61.5	17.6	18.7

**Table 2:** Effect of the Ru composition (1.0–2.0 wt.% Ru-CZO) on the DME conversion and the selectivity of H_2_, CO, CO_2_, and CH_4_ at 750 °C. Operation parameters: DME:O_2_ = 2:1 (vol%); DME:O_2_:N_2_ = 30:15:55 (vol%); and GHSV = 10,000 h^−1^.

Ru (wt.%)	DME conv. (%)	H_2_ sel. (%)	CO sel. (%)	CO_2_ sel. (%)	CH_4_ sel. (%)
1.0	100	66	62	17	21
1.5	100	78	76	14	12
2.0	100	82	80	12	8

## Data Availability

The data that support the findings of this study are available from corresponding author upon reasonable request.
